# Advancing Risk-Based Approaches in Blood Pressure Management: Reflections on the 2025 AHA/ACC Statement

**DOI:** 10.1161/HYPERTENSIONAHA.125.25564

**Published:** 2025-08-28

**Authors:** Kazem Rahimi, Milad Nazarzadeh, Anthony Rodgers

**Affiliations:** Deep Medicine, Nuffield Department of Women’s and Reproductive Health, University of Oxford, United Kingdom (K.R., M.N.).; The George Institute for Global Health, University of New South Wales, Sydney, Australia (A.R.).

**Keywords:** blood pressure, cardiology, cardiovascular diseases, hypertension, risk stratification

Blood pressure (BP) management strategies have evolved substantially over recent decades, shifting from approaches based exclusively on BP thresholds to the adoption of multidimensional, risk-based decision making.^1^ Within this context, the 2025 American Heart Association/American College of Cardiology (AHA/ACC) Scientific Statement on the use of risk assessment in BP management^2^ represents a further welcome advancement towards aligning clinical practice with contemporary evidence. The Statement offers a robust and evidence-informed framework for risk-based BP management. However, in our view, some key issues remain that are worth highlighting.

## Gauging the Impact of the Recommended Risk-Based Strategy

Echoing, to some extent, the 2024 European Society of Cardiology guidelines,^3^ the AHA Statement categorically exempts individuals with BP ≥140/90 mm Hg (stage 2 hypertension) from formal risk assessment. The same exemption is extended to the BP range of 130 to 139/80 to 89 mm Hg (stage 1 hypertension) for those with established cardiovascular disease, diabetes, chronic kidney disease, or any individual with ongoing antihypertensive therapy. These groups are automatically designated as high risk.

**Figure 1.**
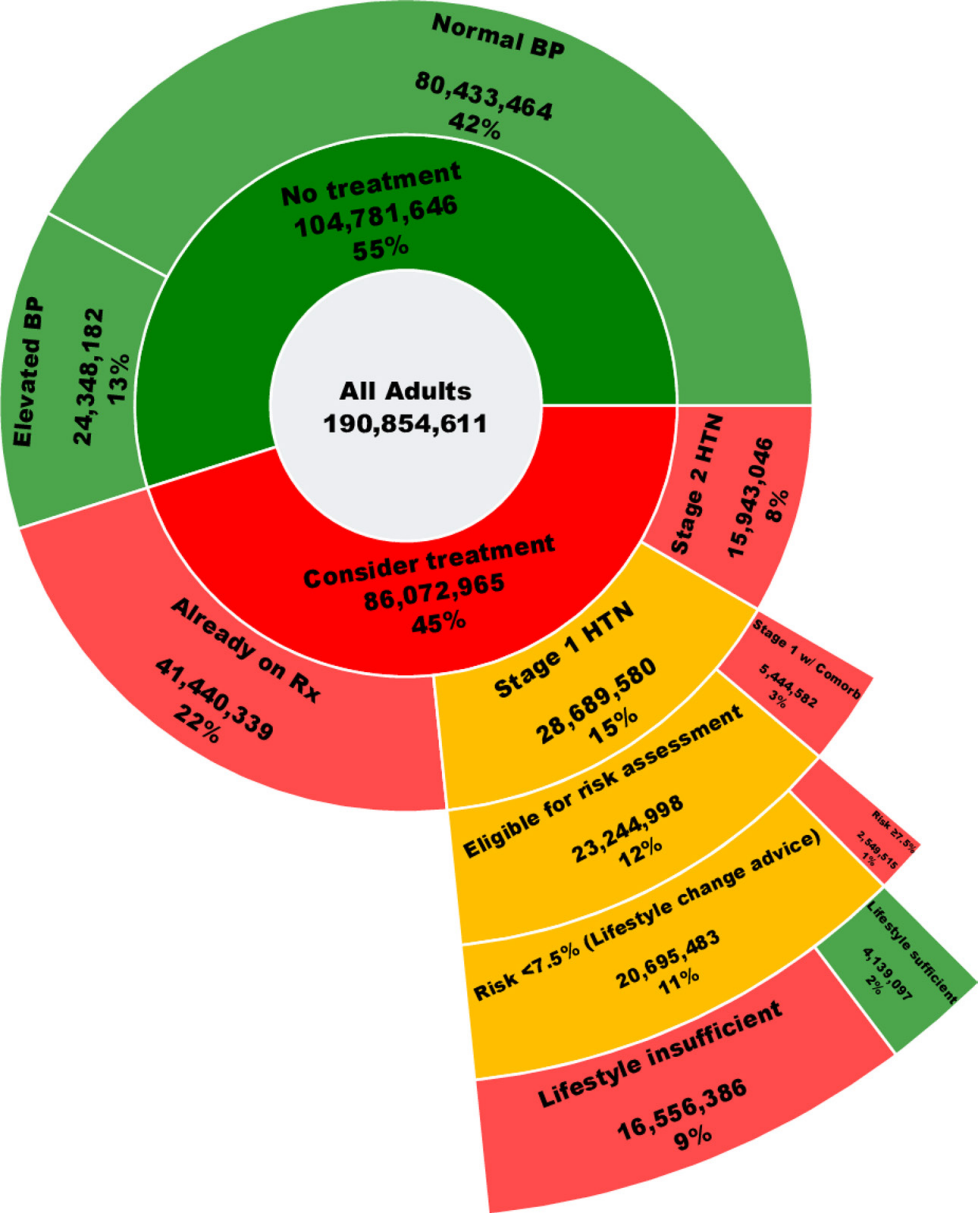
**Proportional distribution of risk-based treatment pathways in the United States adult population according to the American Heart Association/American College of Cardiology (AHA/ACC) 2025 risk-based guideline for treatment of hypertension.** Comorb denotes major comorbidities including established cardiovascular disease, diabetes, or chronic kidney disease, and risk denotes predicted 10-year risk of cardiovascular diseases using the Predicting Risk of Cardiovascular Diseases Events model. Green color depicts those not recommended for pharmacological therapy, amber depicts those subject to formal or informal risk assessment, and red is those subject to pharmacological therapy. Link to interactive plot: https://miladnazarzadeh.github.io/AHA_2025-_risk_assessment_for_BP/Final.html. BP indicates blood pressure; HTN, hypertension; and Rx, antihypertensive medications/prescriptions.

With these exemptions, a formal risk assessment strategy applies to ≈23 million individuals, of whom around 2.5 million (equivalent to 1% of US adults) are projected to exceed the PREVENT risk threshold of >7.5% for immediate pharmacological intervention (Figure 1). These rates are roughly consistent with the application of the European Society of Cardiology guidelines to European adults, where ≈1% to 4% would be recommended for treatment based on their predicted high cardiovascular risk and moderate BP elevation.^4,5^

However, the AHA/ACC statement diverges from the European Society of Cardiology Guidelines in one critical aspect—namely, the management of low-risk individuals. Application of the PREVENT tool would classify ≈21 million people, representing 11% of the US adult population, as low-risk (estimated 10-year risk ≤7.5%). The statement recommends pharmacological treatment in this low-risk group after 3 to 6 months, unless lifestyle modification reduces BP to <130/80 mm Hg. Evidence suggests that a modest proportion of such individuals—approximately one-fifth^6^ —will achieve a BP threshold of <130/80 mm Hg through lifestyle modification. Consequently, the recommended risk-based strategy would result in immediate treatment for 1% of adults (those with stage 1 hypertension and a 10-year risk ≥7.5%) and a trial of lifestyle modification for 11% of adults (those with stage 1 hypertension and a 10-year risk <7.5%), with the majority of the latter group ultimately receiving treatment after 3 to 6 months. This stands in stark contrast to the 33% of the hypertensive population already receiving antihypertensive therapy or recommended for therapy without formal risk assessment and highlights the very limited consequence of risk stratification in practice compared with rule-of-thumb risk factor–based decision making. The small weight of formal risk estimation on treatment decisions is illustrated in Figure 1, where among individuals with stage 1 hypertension, only the small outer green segment (representing 2% of the population) would be deselected from pharmacological treatment.

## Spectrum of Risk and Absolute Risk Reductions in Groups Not Recommended for Risk Quantification

On the contrary, considering the exempted group as uniformly high-risk oversimplifies their risk profiles and introduces the potential for undertreating the highest risk. This is important given the increasing number of therapeutic options and the importance of targeting the most intensive therapy to those at especially high risk. There is substantial heterogeneity of risk profiles among those who are recommended by the ACC/AHA for treatment without formal risk assessment. For example, in a large-scale, population-based study from England, the rate of cardiovascular disease among individuals with systolic BP of 140–150 mm Hg varied by nearly 10-fold.^7^ Conversely, across strata defined by predicted cardiovascular risk, wide variation in baseline BP exerted a modest influence on cardiovascular event rates.^7^ Categorizing these risk levels can be done quite reliably–multivariable risk prediction is less susceptible to variability arising from measurement errors in individual risk factors and may provide a more reliable estimate of overall risk, particularly when repeated measurements of these risk factors are challenging or unavailable.^8^

Patients with preexisting conditions, such as diabetes, are also heterogeneous in terms of cardiovascular risk, making risk stratification clinically valuable for more targeted therapeutic interventions. For example, in a recent study, TRisk—a novel artificial intelligence model developed using routine electronic health records—successfully identified approximately one-quarter of patients with diabetes at low cardiovascular risk, thereby enabling their deprioritization for intensive therapy without compromising the prevention of avoidable events.^9^ Although broad categorization is convenient, it potentially overlooks interindividual variation within these *high-risk* groups. Hence, a formal estimation of risk across all populations—even those with comorbidities—could better select those who are most likely to benefit from treatment than a simpler classification based on BP or disease history.

## Limited Evidence on Treatment Effects in Very Low-Risk Individuals

**Figure 2.**
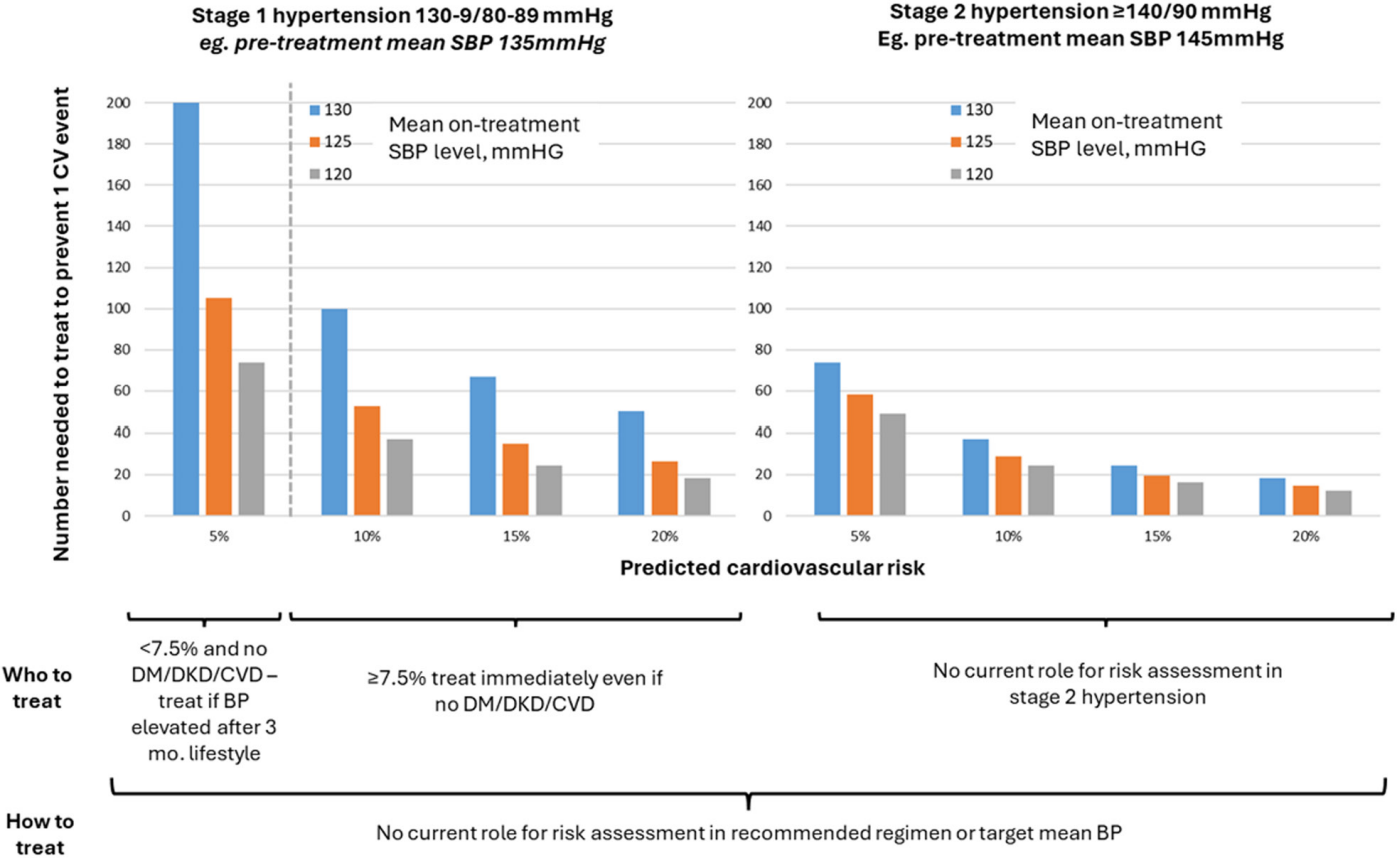
**Number needed to treat (NNT) to prevent 1 cardiovascular event, by hypertension stage, predicted cardiovascular risk and amount of blood pressure (BP) lowering.** Absolute benefits estimated by product of predicted cardiovascular risk and 1-RR: RR=0.90^SBP_diff/5^, where SBP_diff =regimen efficacy, based on 5 mm Hg systolic blood pressure (SBP) reduction conferring RR, 0.90 (BPLTTC 2021). CV indicates cardiovascular; and RR, relative risk.

Another issue with the AHA statement is the extension of pharmacological treatment to those with an estimated 10-year risk of ≤7.5. The evidence for this recommendation is largely based on meta-analyses of BP-lowering trials, which have shown similar relative effects across a range of predicted cardiovascular disease (CVD) risk.^10,11^ However, in the referenced studies, the lowest risk category still had a 10-year CVD risk of about 10%. Direct evidence on the beneficial or harmful effects of BP-lowering therapy on clinical outcomes in low-risk individuals is currently lacking. Although the AHA/ACC Committee acknowledges this lack of evidence with a weaker recommendation for medical treatment (class 2b), it still raises a question around treatment efficiency. Treatment thresholds are most effectively set to target those with clear net clinical benefit, which will involve more intensive treatments being provided to those at higher risk. Treating low-risk individuals will inevitably result in smaller absolute risk reductions and higher numbers needed to treat, with potential implications for health care costs and patient motivation. This is illustrated in Figure 2 for hypothetical patients with differing predicted CVD risk, baseline BP, and expected BP reduction following therapy. In one scenario, an individual with stage 1 hypertension and a predicted CVD risk of 5% would, under the new guidelines, be recommended pharmacological treatment after 3 to 6 months of lifestyle modification. For such an individual, even with intensive BP-lowering therapy reducing systolic BP from 135 to 120 mm Hg, the numbers needed to treat to prevent 1 CVD event would be ≈70. A more modest BP reduction to 130 mm Hg would result in a numbers needed to treat of around 200. This illustrates that stratifying treatment decisions by both predicted risk and the anticipated intensity of BP reduction could facilitate more targeted identification of individuals most likely to benefit from therapy.

## Trade-Offs Between Simple and Parsimonious Versus More Complex Prediction Models

The AHA statement endorses the Predicting Risk of Cardiovascular Disease Events (PREVENT) model as the primary tool for cardiovascular risk prediction in BP management. The key novel aspect of PREVENT is that it includes both atherosclerotic CVD and heart failure as its predicted outcomes. Although the inclusion of heart failure is intended to capture broader morbidity, it is important to recognize that the risks of atherosclerotic CVD and heart failure are highly correlated (Pearson R>0.9).^12^ This is also unsurprising given their shared risk factors. Indeed, the PREVENT tool incorporates mostly the same predictors as used in existing models, such as the Pooled Cohort Equations, with only minor additions, including body mass index and socioeconomic status. Consequently, their discriminatory performance is also comparable.

Important arguments put forward in favor of PREVENT include its representativeness of a broader contemporary US population and its ease of adoption within the US health care system. These are valid considerations; however, in the absence of a material improvement in discriminatory performance, it could be argued that an existing, more parsimonious model might have sufficed to achieve these aims. Indeed, one of the key barriers to the uptake of cardiovascular disease risk prediction models is their potential to promote overtreatment at the individual level—an issue that can only be mitigated through demonstrably higher predictive performance.^9^ Nevertheless, PREVENT offers several practical advantages: it is designed for use with routinely collected electronic health record data, making it potentially scalable and readily implementable within the existing infrastructure of the US health care system.

Future developments may involve the adoption of more complex multimodal deep learning models, which have shown potential for superior predictive accuracy in some studies.^9^ However, such models require rigorous validation and careful integration into clinical workflows. Until this is achieved, national models such as PREVENT—with the potential for calibration across subpopulations—represent a pragmatic compromise between sophistication and feasibility.

## Future Outlook

Looking ahead, we propose a substantially expanded role for risk-based treatment, extending its use to all individuals with elevated BP, not only to inform who should be treated but also to guide how treatment should be tailored. This approach aligns naturally with the central aim of risk assessment: to direct pharmacological therapy—along with its attendant risks and costs—towards those at highest risk. Higher risk individuals are most likely to achieve greater absolute benefits, thereby maximizing efficiency in reducing cardiovascular disease risk (ie, lowering the number needed to treat). Among higher-risk individuals, however, the magnitude of risk remains important and should directly inform treatment decisions. Figure 2 illustrates that, for individuals with identical baseline BP, the benefits of achieving a mean on-treatment systolic BP of 120, 125, or 130 mm Hg are largely determined by their underlying cardiovascular risk.^13^ This matters because the rates of adverse effects and associated costs can vary so widely across treatment regimens; for example, monotherapy with an angiotensin II receptor blocker is inexpensive and is associated with fewer adverse effects than placebo.^14^ Whereas maximal therapy with 3 to 4 maximum-dose agents plus renal denervation entails a significantly higher adverse effect and cost profile. With a wave of novel therapies on the horizon, risk stratification among individuals at elevated risk will become increasingly important from a cost-effectiveness perspective as well.

According to the 2017 Guideline, 68 million individuals had uncontrolled hypertension and were recommended for antihypertensive therapy; of these, 35 million were untreated, and 33 million were receiving treatment but had not attained target BP levels. We advocate for the application of risk stratification to guide priority interventions in both cohorts, informing optimal escalation strategies for those inadequately controlled, and prioritizing interventions to address nontreatment in the untreated population. Drawing a parallel with lipid-lowering therapy, it is both rational and evidence-based to ensure that individuals at the highest risk receive the most effective—and, where appropriate, more intensive—therapeutic regimens. We propose that BP-lowering interventions should be classified by intensity, analogous to lipid-lowering therapies: low, moderate, and high intensity, corresponding to reductions in systolic BP of <10, 10 to 19, and ≥20 mm Hg, respectively.^15^ Those at the highest absolute risk should generally be prioritized for high-intensity regimens, provided adverse effects do not preclude their use. Achieving a mean SBP of ≤120 mm Hg is essential to maintain the majority of BP readings below 130/80 mm Hg over time, given the natural intraindividual variability in BP. Thus, all patients with untreated mean SBP >140 mm Hg will require high-intensity regimens to attain and sustain optimal BP control and minimize cardiovascular risk. The substantial expansion of high-intensity therapy should therefore be directed primarily towards individuals at the highest absolute risk and with the greatest gap to achieving optimal BP. Given the heightened urgency in patients with high predicted risk, immediate pharmacological intervention—potentially with dual therapy—should be considered as the default strategy. Naturally, this approach must be tailored to patient preferences and tolerability; nonetheless, the evidence increasingly supports early, decisive intervention in those most likely to benefit.

## Conclusions

Risk-based strategies are poised to redefine cardiovascular prevention in the future, shifting the focus from arbitrary BP thresholds to holistic risk-harm stratification, and enabling a framework with more intelligent, efficient, and equitable care. The AHA/ACC 2025 statement marks meaningful progress, yet further refinement is essential. Broader application of risk models—including in populations with comorbidities and elevated BP—would fully realize the potential of this approach. Treatment protocols should also evolve to incorporate both predicted risk and the anticipated magnitude of BP reduction. High-risk patients warrant prompt, evidence-based intervention, including early consideration of dual therapy, to maximize the benefits of BP-lowering treatment.

Ultimately, realizing the promise of risk-based prevention will require ongoing innovation in model development and its implementation into routine practice. The path forward is unmistakable: smarter, risk-tailored prevention is both the future and the imperative for cardiovascular disease management in the 21st century.

## ARTICLE INFORMATION

### Sources of Funding

None.

### Disclosures

None.
